# Beneficial effects of levobupivacaine regional anaesthesia on postoperative opioid induced hyperalgesia in diabetic mice

**DOI:** 10.1186/s12967-015-0575-0

**Published:** 2015-07-02

**Authors:** Anne Gomez-Brouchet, Nelly Blaes, Lionel Mouledous, Olivier Fourcade, Ivan Tack, Bernard Francès, Jean-Pierre Girolami, Vincent Minville

**Affiliations:** Service d’Anatomie Pathologique et Histologie-Cytologie, IUCT Oncopôle, 1 Avenue du Juliot Curie, 31059 Toulouse Cedex 9, France; Department of Anaesthesiology and Intensive Care, Toulouse University Hospital, 31432 Toulouse, France; CNRS, IPBS, Institut de Pharmacologie et de Biologie Structurale, 205 Route de Narbonne, 31077 Toulouse, France; Institute of Metabolic and Cardiovascular Diseases, I2MC, INSERM, U1048, Université Paul Sabatier, 31432 Toulouse, France; Université de Toulouse, Centre de Recherches sur la Cognition Animale, CNRS, UMR 5169, Université Paul Sabatier, 118 Route de Narbonne, 31062 Toulouse, France

**Keywords:** Diabetes, Levobupivacaine, Naloxone, Opioid induced hyperalgesia, Pain, Plantar incision, Regional anaesthesia, Sufentanil

## Abstract

**Background:**

Diabetic neuropathy is one of the most common complications of diabetes and causes various problems in daily life. The aim of this study was to assess the effect of regional anaesthesia on post surgery opioid induced hyperalgesia in diabetic and non-diabetic mice.

**Methods:**

Diabetic and non-diabetic mice underwent plantar surgery. Levobupivacaine and sufentanil were used before surgery, for sciatic nerve block (regional anaesthesia) and analgesia, respectively. Diabetic and non-diabetic groups were each randomly assigned to three subgroups: control, no sufentanil and no levobupivacaine; sufentanil and no levobupivacaine; sufentanil and levobupivacaine. Three tests were used to assess pain behaviour: mechanical nociception; thermal nociception and guarding behaviours using a pain scale.

**Results:**

Sufentanil, alone or in combination with levobupivacaine, produced antinociceptive effects shortly after administration. Subsequently, sufentanil induced hyperalgesia in diabetic and non-diabetic mice. Opioid-induced hyperalgesia was enhanced in diabetic mice. Levobupivacaine associated to sufentanil completely prevented hyperalgesia in both groups of mice.

**Conclusion:**

The results suggest that regional anaesthesia can decrease opioid-induced hyperalgesia in diabetic as well as in non-diabetic mice. These observations may be clinically relevant for the management of diabetic patients.

## Background

Diabetes affected approximately 387 million people worldwide in 2014 (International Diabetes Federation, http://www.idf.org/diabetesatlas); by 2035 these numbers are projected to rise up 592 million people [1–3]. Neuropathy, defined as hypersensitivity to noxious stimuli, is a common complication of diabetes which appears in about 60% of the diabetic patients [4, 5]. Since relief of pain in diabetic patients improves the functional and health status outcomes [6], treatment of painful diabetic neuropathy is essential. Anaesthetic and analgesic management of these patients is still challenging for anaesthesiologists [7].

Perioperative pain is a risk factor for developing chronic pain after surgery. Exogenous opioids provide analgesia to acute pain; however, opioid-induced hyperalgesia (OIH) may subsequently occur with a paradoxical increase in pain after opioid administration. OIH has been clearly evidenced in patients after surgery, in particular when exposed to high doses of the widely perioperatively used selective μ opioid receptor agonist remifentanil [6, 8, 9].

Diabetic patients have additional risks in surgery, with more instable cardiovascular control under anaesthesia [10] and loss of myocardial preconditioning of remifentanil [11]. Moreover, sustained analgesic action can be altered in diabetic patients [6]. Recently, peripheral nerve block has become a popular anaesthetic option in the perioperative management of patients with diabetes mellitus (types 1 and 2) [12]. Regional anaesthesia (RA) provides better postoperative analgesia than does general anaesthesia, while avoiding the cardiopulmonary and insulin-resistance effects of general anaesthesia [7].

Rodent models of diabetes (induced by streptozocin injection) develop peripheral neuropathy and slower conduction times in both sensory and motor nerves (by 5 weeks) [13, 14]. These changes in peripheral nerves may affect the onset of a local anaesthetic block, and even the susceptibility to nerve toxicity of local anaesthetics [7, 15]. Recently, it has been shown that the perioperative use of regional anaesthesia reduced postoperative hyperalgesia and inflammation in rats [16]. OIH has been first evidenced in animal models [17, 18]. Remifentanil induced pro-nociceptive effects in a model of plantar incision pain in mice [19]. Rodent models are currently used to study underlying mechanisms and new therapeutics. The aim of the present study was to evaluate the impact of regional anaesthesia (levobupivacaine sciatic block) on postoperative (plantar surgery) sufentanil induced hyperalgesia in diabetic and non-diabetic mice, focusing primarily on hyperalgesia and secondarily on wound healing.

## Methods

### Animals

Mice were housed in a pathogen-free facility and handled in accordance with the procedures outlined in the Council Directive 86/609/EEC. The investigation conformed to the Guide for the Care and Use of Laboratory Animals published by the US National Institute of Health (NIH publication No. 85-23, revised 1996). Experimental procedures were conducted according to the official edict presented by the French Ministry of Agriculture (Paris, France) and the recommendations of the Helsinki Declaration. The French Animal Care and Use Committee of the Toulouse University approved these experiments. Male 10–12 week-old C57BL/6J mice (Janvier, France) weighing 22.7–25.0 g were used. After arrival, animals were allowed to recover for 1 week in groups of six animals in plexiglas cages. During the whole experiment, mice were housed at 21°C under a controlled 12 h light dark cycle and received water and food ad libitum. At end point, mice were killed with compressed carbon dioxide and the paws were removed and rapidly processed for histological analysis.

### Induction of diabetes

Streptozotocin (STZ)-treated mice were used as a model of type 1 diabetes [20]. Diabetes was induced by three consecutive intraperitoneal injections of STZ (Sigma-Aldrich, St-Quentin-Fallavier, France), at a daily dose of 80 µg/g body weight in 100 µl of sterile 0.05 M sodium citrate (pH 4.5). The non-diabetic control group received an equal volume of sodium citrate vehicle. Glycaemia was assessed using an Accu-chek glucometer (Roche, Paris, France) on blood from the tail vein. Mice with blood glucose level above 400 mg/dl were considered diabetic and included in the experiments. Experiments started 41 days after the last STZ injection to allow development of diabetic neuropathy [14].

### Surgery

The procedure for plantar incision has been described in previous report from Brennan’s team [21]. Briefly, placing the animals in a plastic box that contained 4% sevoflurane in air induced anaesthesia. The depth of anaesthesia was assessed using withdrawal reflex of the left paw (the forelimb pedal withdrawal response after a hard pinched toe was absent). Anaesthesia was maintained by administrating 3–4% sevoflurane in air via a conenose. Animals received or not levobupivacaine before the incision as described below in the experimental groups section. After antiseptic preparation of the right hind paw with povidone iodine, a deep 8-mm longitudinal incision was made with a number 11 blade through the skin, fascia, and (plantar flexor digitorum brevis) muscle of the right hind paw [22]. The skin was closed with two single 6-0 nylon sutures, and the wound site was covered with povidone iodine. After surgery, anaesthesia was discontinued, and the animals were allowed to recover in their cage.

### Sciatic nerve block and criteria for nerve block assessment

Regional anaesthesia (RA) of the sciatic nerve was performed as follows. The sciatic nerve was identified using a nerve stimulator settled at 0.2 mA and 1 Hz (B-Braun, Melsungen, Germany) via a 22 G-diameter needle [23]. After localization of the sciatic nerve just posterior of the great trochanter, each animal received a single 0.1 ml injection of 5% levobupivacaine (Chirocaïne, Abbott, Rungis, France) in the sciatic nerve area. Efficacy of the RA was assessed by the loss of motor control in the injected limb according to the scores: 0 (normal movement); 1 (unable to flex the limb completely); and 2 (total paralysis) [23]. Any animal unable to walk normally with the injected limb was considered to have a positive response to RA. Efficacy of the motor blockade was evaluated every 5 min up to the end of the sciatic nerve blockade. Ten diabetic mice were compared with ten non-diabetic mice. These tested mice were not included in the following experimental groups. To evaluate RA in mice of the experimental groups, only mice with efficient block (total paralysis) were included.

### Experimental groups

Non-diabetic and diabetic mice were randomly assigned each to three different groups (groups 1–3 and groups 4–6, respectively) with 10 mice per group: control (groups 1 and 4: no OIH, no sciatic block, plantar incision), OIH (groups 2 and 5: OIH, no sciatic block, plantar incision), and OIH + RA (groups 3 and 6: OIH, sciatic block, plantar incision). We studied the early and long-lasting effects of sufentanil on nociceptive threshold using a procedure designed to partly mimic its use in surgery. More precisely, in the C control groups (1 and 4); saline was subcutaneously injected four times at 15-min intervals. Surgery was performed as described above after the last injection. In the S groups (2, and 5), sufentanil was injected four times at 15-min intervals (10 µg/kg per subcutaneous injection), resulting in total doses of 40 µg/kg [24]. Surgery was performed as described above after the last injection. In the S + RA groups (3 and 6), sufentanil was injected four times (10 µg/kg per injection, subcutaneously) at 15-min intervals, resulting in total doses of 40 µg/kg. [24] Sciatic blockade was performed before the last sufentanil injection. Plantar surgery was performed as described above after the last injection. Pain testing (mechanical stimulation, hot plate test, and guarding behaviors) was performed before the surgery (D-1 and D0: basal B) and post surgery (30 min, 2, 4 and 6 h after surgery, once daily until D7, and at D14). In addition, to unmask OIH, for all groups of mice on day D7, a single subcutaneous injection of the non selective opioid antagonist naloxone (naloxone hydrochloride, Sigma) was performed (1 mg/kg in 0.9% NaCl) [25] and the mice were tested for mechanical stimulation and hot plate before and 30 min after naloxone injection (BN, AN time points, respectively).

### Behavioral tests

Mechanical nociception (allodynia) was quantified by measuring the hind paw withdrawal response to von Frey filament stimulation. Unrestrained mice were placed beneath a clear plastic chamber on an elevated mesh floor and were allowed to acclimate. Withdrawal responses to mechanical stimulation were determined using calibrated von Frey filaments applied from underneath the cage through the mesh floor to the hindpaw plantar skin adjacent but avoiding the wound. The filament was pushed until it slightly bowed and was maintained in that position for 6 s. Each von Frey filament was applied once, starting from least (0.008 g) to greatest forces until a withdrawal response was reached which was considered a positive response [26]. The entire test was repeated three times. The lowest positive force from the three tests was considered as the withdrawal threshold (data expressed in g) [27, 28].

Thermal nociception was assessed by the withdrawal response to thermal stimulus using a modified hot-plate test [24, 29]. The time to hind paw withdrawal from a 52°C hot plate (thermal withdrawal latency) was measured. The paw was removed from the plate by the investigator after a maximal 12 s time to avoid thermal injury and thermal hyperalgesia [27–29].

Guarding behaviours (non-evoked pain behaviours) were assessed using a cumulative pain score as described by Banik and Brennan [30]. Unrestrained mice were placed beneath a clear plastic chamber on an elevated mesh floor and were allowed to acclimate. Animals were habituated to the testing scenario by two testings. Testings were all performed in the same cage in a quiet room and by the same experimenter who was blinded with respect to the treatment groups. Both paws of each animal were closely observed during a 1-min period repeated every 5 min for 1 h. Depending on the position in which each paw was found during the majority of the 1 min scoring period, a 0, 1, or 2 was given. Full weight bearing of the paw (score = 0) was present if the wound was blanched or distorted by the mesh. If the paw was completely off the mesh, a score of 2 was recorded. If the area of the wound touched the mesh without blanching or distortion, a 1 was given. The sum of the 12 scores (0–24) during the 1 h session was obtained for each paw. The difference between the scores from the incised paw and non-incised paw was the cumulative pain score for that 1 h period.

### Histopathological examination of skin incision and wound healing

The histological examination of skin incision and wound healing of mice was performed for three mice in each group of diabetic and non-diabetic mice, at day 1 (D1), D7 and D14. Paw specimens were fixed in formalin and embedded in paraffin. Decalcification was performed if necessary. Sections of 4 µm thickness were stained with hematoxylin and eosin. To assess the effect of RA on wound healing, histological examination was performed at D14 in the diabetic and non-diabetic mice. The pathologist was blinded for the experimental group.

A pathologist experienced in this field who was blinded to the animal’s group, using light microscopy at 10× and 40× magnifications by randomly sampling the preparation, conducted the histopathological examination. Inflammation infiltration was analysed by four-grade semi quantitative scoring: 0, no inflammation, 1, weak, 2 moderate, 3 strong.

### Statistical analysis

Data are expressed as mean ± SD. The values of behavioural testing were not normally distributed and thus were analysed non-parametrically. To assess whether the withdrawal responses changed over time, Friedman’s test was used. When Friedman’s test was significant (*P* < 0.05), pairwise comparisons were performed using Wilcoxon’s signed rank test. Time points comparisons between groups were performed using first a non-parametric Kruskal–Wallis test; when Kruskal–Wallis test was significant (*P* < 0.05), pairwise comparisons were performed using the Mann–Whitney U test.

## Results

### Induction of diabetes in mice

Fifty mice with initial mean body weight of 25.5 ± 3.0 g were treated with three consecutive STZ i.p injections. Twelve days after the injections, 10 of the 50 STZ-treated mice did not develop diabetes, as indicated by sub-normal glycemia or mild hyperglycemia (<400 mg/dl); these animals were excluded from the following experiments. Mean parameters for the diabetic mice were glycemia 520 ± 80 mg/dl, hematocrit 48.3 ± 2.6%, mean body weight 23.4 ± 3.2 g. Experiments started 41 days after STZ treatment, with thirty STZ-treated stably diabetic well-walking mice (glycemia 610 ± 130 mg/dl, hematocrit 47.9 ± 2.2%, mean body weight 26.4 ± 2.9 g) and thirty vehicle-treated non diabetic mice (glycemia 133 ± 41 mg/dl, hematocrit 49.9 ± 2.1%, mean body weight 28.7 ± 2.3 g).

### Effect of diabetes on duration of sciatic nerve blockade in mice

**Figure 1:**
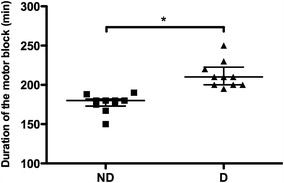
Effect of diabetes on the duration of regional anaesthesia in mice. The duration of a sciatic nerve block with levobupivacaine was evaluated in non-diabetic and diabetic mice. The duration of loss of motor control (min) was recorded for each animal. *Min* minutes, *ND* non-diabetic mice, *D* diabetic mice, n = 10 per group. *P < 0.05.

The duration of the sciatic nerve blockade (regional anaesthesia, RA) was significantly longer in the diabetic mice as compared to the non-diabetic mice (Figure 1).

### Effect of regional anaesthesia on opioid induced hyperalgesia in non-diabetic mice

**Figure 2:**
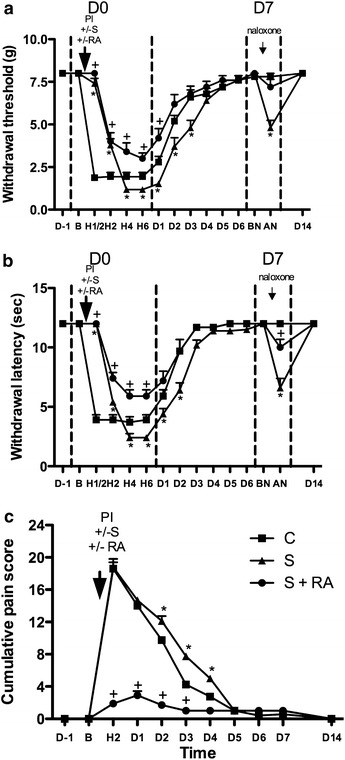
Effect of regional anaesthesia on opioid induced hyperalgesia in non-diabetic mice. The actions of levobupivacaine sciatic nerve block on perioperative sufentanil induced hyperalgesia were evaluated by three behavioural tests: **a** time course of mechanical allodynia (withdrawal threshold to von Frey filament, in g), results expressed as mean ± SD; **b** time course of heat hyperalgesia (withdrawal latency on hot plate, in s), results expressed as mean ± SD; **c** guarding pain behaviour (0–24 cumulative pain score). Non-diabetic control group 1 (C): no sufentanil, no levobupivacaine sciatic block, plantar incision; non-diabetic OIH group 2 (S): sufentanil, no levobupivacaine sciatic block, plantar incision; non-diabetic OIH + RA group 3 (S + RA): sufentanil, levobupivacaine sciatic block, plantar incision. Naloxone injection was performed for all groups on D7. *P < 0.05 compared group 2 to group 1 (control); ^+^ *P*<0.05 compared group 3 to group 1. *AN* after naloxone, *BN* before naloxone, *B* basal, *C* control, *D* day, *H* hour, *g* gram, *PI* plantar incision, *RA* regional anaesthesia, *S* sufentanil, *s* second.

No differences in the measured parameters were observed between the non-diabetic groups before experiments (D-1) (Figure 2). At D0 shortly after administration (H ½), sufentanil produced antinociceptive effects with modifications of the responses to mechanical and thermal stimulations, and of the subjective pain scale (Figure 2). And then sufentanil induced hyperalgesia (OIH) from D0-H4 to D3, as regards mechanical nociception, thermal nociception and guarding score (p < 0.05 between the H2-D3, H2-D2, D3-D5 points of the S and C curves in Figure 2a–c, respectively). At D0, the sufentanil and RA association produced antinociceptive effects shortly after administration (H ½), similarly to sufentanil alone (Figure 2). RA associated to sufentanil prevented the D0-H4 to D3 OIH, when compared to the sufentanil group (Figure 2). The pain parameters were even improved in the S + RA group compared to the control C group (P < 0.05 between the H2–H6 points of the S + RA and C curves in Figure 2a–c respectively). The results suggest that RA prevented OIH in non diabetic mice.

### Effect of regional anaesthesia on opioid induced hyperalgesia in diabetic mice

**Figure 3:**
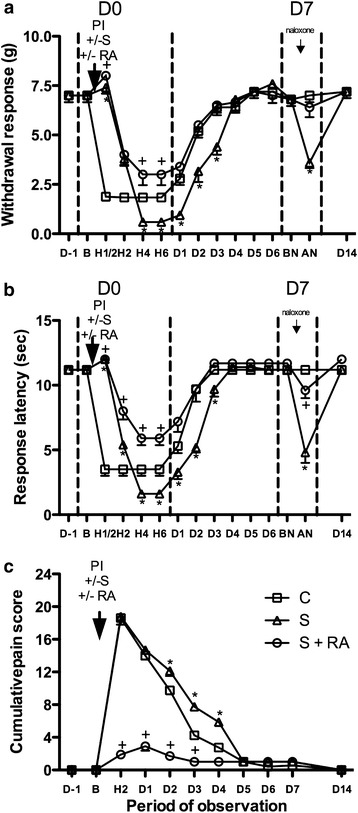
Effect of regional anaesthesia on opioid induced hyperalgesia in diabetic mice. The actions of levobupivacaine sciatic nerve block on perioperative sufentanil induced hyperalgesia were evaluated by three behavioural tests: **a** von frey; **b** hot plate; **c** guarding pain behaviour. Diabetic control group 4 (C): no sufentanil, no levobupivacaine sciatic block, plantar incision; diabetic OIH group 5 (S): sufentanil, no levobupivacaine sciatic block, plantar incision; diabetic OIH + RA group 6 (S + RA): sufentanil, levobupivacaine sciatic block, plantar incision. *P < 0.05 compared group 5 to group 4 (control); ^+^ *P*<0.05 compared group 6 to group 4. *AN* after naloxone, *BN* before naloxone, *B* basal, *C* control, *D* day, *H* hour, *g* gram, *PI* plantar incision, *RA* regional anaesthesia, *S* sufentanil, *s* second. Data presented as mean ± SD.

No differences in the measured parameters were observed between the diabetic groups before experiments (D-1) (Figure 3). However, basal withdrawal thresholds (D-1) and latency were slightly reduced, when compared to parameters of the non-diabetic mice at D-1 (Figure 2a, b) (*P* < 0.05). As for the non-diabetic mice, at D0, sufentanil produced antinociceptive effects shortly after its administration (H ½) with modification of the responses to mechanical and thermal stimulation, as well as the subjective pain scale (Figure 3). Then sufentanil induced hyperalgesia (OIH) from D0-H4 to D3 as regards mechanical nociception, thermal nociception and guarding score (P < 0.05 between the H4-D3, H2-D3, D2–D4 points of the S and C curves in Figure 3a–c, respectively) reaching lower nociceptive threshold values than in non-diabetic mice (Compare Figure 3 to Figure 2). At D0, sufentanil and RA association produced antinociceptive effects shortly after administration (H ½), similarly to sufentanil alone (Figure 3). Then RA associated to sufentanil prevented the D0-H4 to D3 OIH when compared to the sufentanil group (Figure 3). The parameters were even improved in the S + RA group compared to the control C group (P < 0.05 between the H4–H6, H2–H6, H2-D3 points of the S + RA and C curves, Figure 3a–c respectively). The results suggest that RA prevented OIH in diabetic mice.

### Effect of regional anaesthesia on histopathological examination of skin incision and wound healing

#### Histology of mice paws

**Figure 4:**
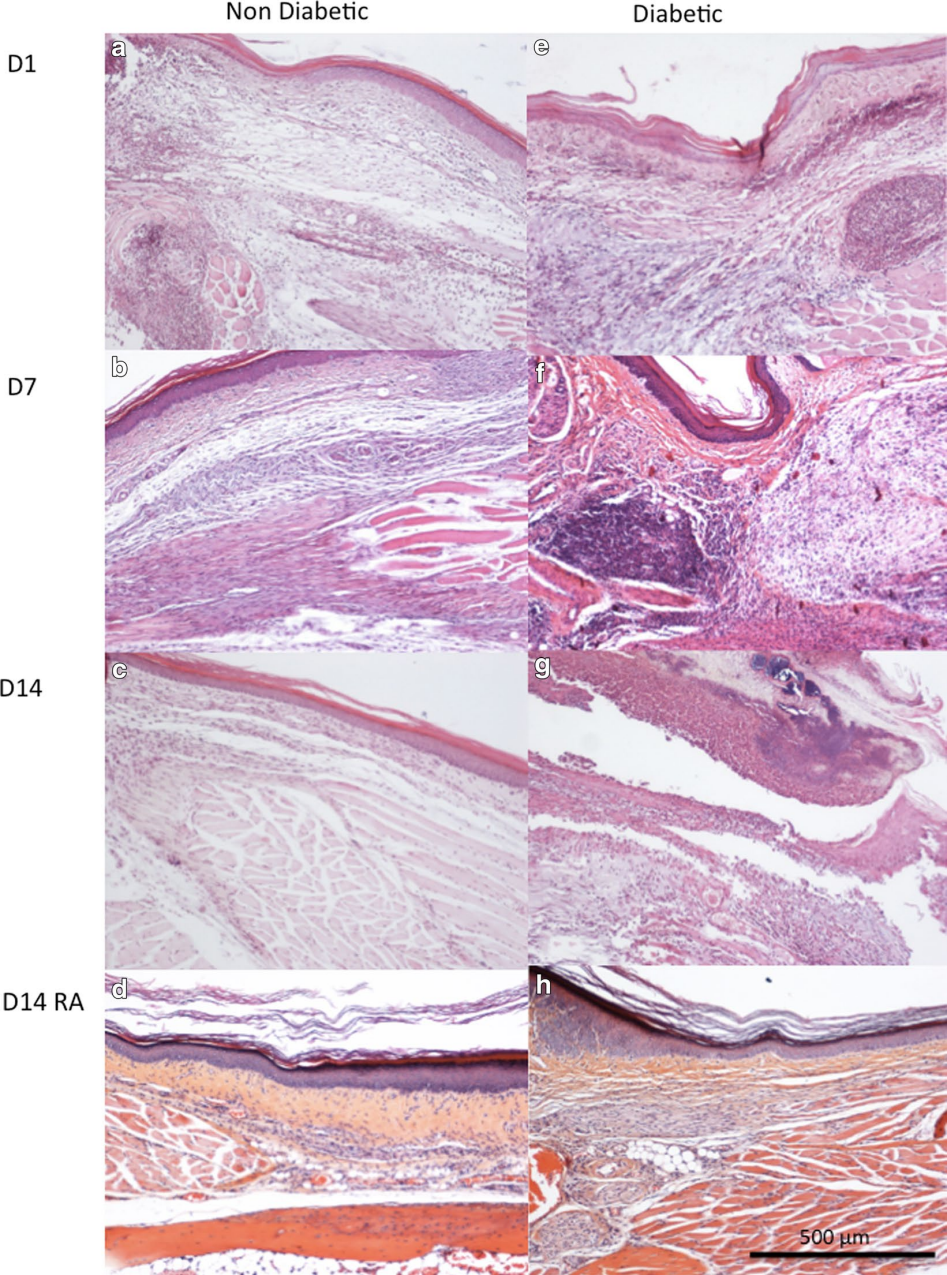
Histology of mouse hindpaws. The effect of levobupivacaine and sufentanil were tested on histopathology of diabetic and non-diabetic mouse hind paws at different days postsurgery: **a** non-diabetic mice 1 day (*D1*) after skin incision showing acute inflammatory infiltration with polynuclear cells and oedema. **b** Non-diabetic mice 7 days (*D7*) after skin incision showing myofibroblastic proliferation with some lymphocyte inflammatory cells. **c** Non-diabetic mice 14 days (*D14*) after skin incision showing wound healing of the conjunctive tissue with only few inflammatory cells and still fibroblastic superficial proliferation. **d** Non-diabetic mice with RA at D14 showing wound healing of the conjunctive tissue with only few inflammatory cells and still fibroblastic superficial proliferation. **e** Diabetic mice at D1 showing acute inflammatory infiltration with polynuclear cells and oedema. **f** Diabetic mice at D7 showing persistent acute inflammatory cells and oedema. **g** Diabetic mice at D14 showing persistent acute inflammatory cells and oedema. **h** Diabetic mice with regional anaesthesia at D14 showing wound healing of the conjunctive tissue with only few inflammatory cells and still fibroblastic superficial proliferation.

Histology of hind paws is shown in Figure 4. Wound healing occurred in non-diabetic mice paws between D1 and D14 (compare Figure 4a–c) whereas persistent acute inflammatory cells with delayed wound healing were observed in diabetic mice paws at D14 (Figure 4e–g). RA did not alter healing in non-diabetic mice at D14 (Figure 4d) whereas it reduced inflammation and improved wound healing in diabetic mice paws (Figure 4h).

#### Regional anaesthesia influences wound inflammation

**Figure 5:**
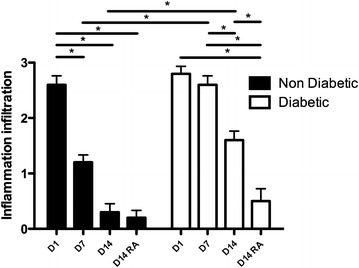
Regional anaesthesia influences wound inflammation. Inflammation infiltration diminished from D1 to D14 in non-diabetic mice. No difference was found at D14 concerning the wound inflammation between non-diabetic with or without regional anaesthesia. No significant difference was found from D1 to D7 in diabetic mice. Inflammation infiltration diminished from D7 to D14 in diabetic mice. Inflammation infiltration was diminished in diabetic mice at D14 when regional anaesthesia was performed. No difference was found at D1 concerning the wound inflammation between non-diabetic and diabetic mice. But, at D7 and D14 inflammation infiltration was higher in diabetic mice when compared to non-diabetic mice. However, when regional anaesthesia was performed no difference was found between diabetic and non-diabetic mice at D14.

Effect of RA on wound infiltration is shown in Figure 5. Inflammation infiltration diminished from D1 to D14 in non-diabetic mice. No difference was found at D14 concerning the wound inflammation between non-diabetic with or without regional anaesthesia. No significant difference was found from D1 to D7 in diabetic mice. Inflammation infiltration diminished from D7 to D14 in diabetic mice. Inflammation infiltration was diminished in diabetic mice at D14 when regional anaesthesia was performed. No difference was found at D1 concerning the wound inflammation between non-diabetic and diabetic mice. But, at D7 and D14 inflammation infiltration was higher in diabetic mice when compared to non-diabetic mice. However, when regional anaesthesia was performed no difference was found between diabetic and non-diabetic mice at D14.

## Discussion

Results from this study provide the first evidence that regional anaesthesia (RA) prevented OIH in diabetic as well as in non-diabetic animals. We used an STZ-induced model of diabetes and found that RA reduced the exaggerated postoperative pain exacerbated by hyperalgesia in diabetic mice. Importantly, RA completely prevented sufentanil-induced hyperalgesia (OIH) in non-diabetic mice as well as in diabetic mice.

The experimental STZ-induced diabetic rodent model is commonly used to investigate diabetic pain [14, 31–33]. In the present study, we used STZ mice with stably established hyperglycemia (5 weeks); in accordance with previous reports, polydipsia and reduced weight occurred after STZ administration. In addition, the mice developed diabetes-induced neuropathy with persistent hyperalgesia (reduced von Frey withdrawal response at D-1), as described in previous reports [31] [34–36].

Local anaesthesia was performed through sciatic nerve blockade with levobupivacaine. We observed increased duration of the sciatic block in the diabetic mice. This first report in diabetic mice is consistent with previous findings in rats [12, 37, 38].

Opioid-induced hyperalgesia (paradoxical opioid-induced pain hypersensitivity) occurred in non-diabetic mice under sufentanil (Figure 2, H2 to D3). OIH also occurred and was even enhanced in diabetic mice; we observed increased sufentanil-induced lowering of von Frey withdrawal threshold and thermal withdrawal latency in diabetic compared to the lowering in non-diabetic mice at D0 H4–6.

Regional anaesthesia with levobupivacaine was efficient in preventing postoperative hyperalgesia and OIH in diabetic mice as well as in non-diabetic mice. This was demonstrated by the complete prevention of sufentanil-lowered withdrawal threshold and shortened response latency during the D0–D4 period; the parameters were even significantly better than the control values without sufentanil (Figures 2 and 3. This is the first evidence that RA can prevent OIH in diabetic models. In our study, perioperative regional anaesthesia was associated to high sufentanil (10 µg/kg). Previously, RA (long term ropivacaine sciatic nerve block) before plantar surgery reduced postoperative pain and long-term pain sensitization in rat [39, 40]. However, the protective effect was lost when high doses of opioid (fentanyl) were used intraoperatively, showing that OIH was not impaired in rats.

The mechanism underlying OIH involves NMDA receptors [40, 41]. Increased NMDA activity also contributes to central sensitization in neuropathic pain, including diabetic neuropathy [42–44]. The mechanisms may also involve mTOR signalling, important in diabetic complications [45], neuropathic pain [46] and morphine hyperalgesia [47]. Very recently, hyperalgesia induced by sufentanil, locally injected in paws, was prevented by an NMDA antagonist, in both diabetic and non-diabetic rats [48]. Here, RA was performed using levobupivacaine, a local anaesthetic, possibly acting also as NMDA antagonist [49–51]. Whether the impact of levobupivacaine on NMDA receptors could be involved in the prevention of OIH was not directly evaluated in the present study.

Wound healing of the incised paw, observed as secondary parameter, appeared improved by RA in the diabetic mice. RA has been reported to impact local inflammation and wound healing with either neutral, negative or positive effects on healing in non-diabetic animals [52]. Normal wound healing consists of haemostasis, inflammation, proliferative phase and fibrotic remodelling. In our study neutrophils infiltration and oedema were similar in diabetic and non-diabetic mice paws at D1 post-surgery. At D7, lymphocyte/plasmocytes/fibroblasts replaced neutrophils in non-diabetic mice whereas neutrophils were still present in diabetic mice. At D14, fibrosis (remodelling with fibroblasts) was observed in non-diabetic mice whereas an inflammatory state was still observed in diabetic mice paws. When RA was performed, no alteration was observed in non-diabetic mice at D14 (remodelling) whereas remodelling was induced in diabetic mice. Bupivacaine has been reported to influence cutaneous wound inflammation (increased neutrophil numbers) in mice while healing (re-epithelialization) was not impaired [53]. In rats, levobupivacaine augmented the fibrotic remodelling [54]. However, despite early beneficial effects, long time levobupivacaine impaired wound healing in rats [52]. Our preliminary histopathological observation is compatible with a positive impact of RA on healing in the diabetic animals (Figures 4, 5).

The present diabetic model exhibits clinical relevance, including diabetic neuropathy [9] and increased sciatic nerve block. In diabetic patients, sciatic block required lower doses [55] and subgluteal sciatic nerve block duration was increased in type 2 diabetes [56]. Anaesthetic and analgesic management of diabetic patients is still challenging. Clinical evidences show that regional anaesthesia might positively impact hyperalgesia and postsurgical pain [7]. OIH has been clearly evidenced in patients after surgery [14]; however the impact of diabetes on postoperative OIH has not yet been specifically studied in patients.

## Conclusion

This study shows that regional anaesthesia can prevent opioid induced hyperalgesia in diabetic mice. These observations may be clinically relevant.

## Abbreviations

OIH: opioid-induced hyperalgesia
PI: plantar incision
RA: regional anaesthesia
STZ: streptozotocin
